# Common and different alterations of bone marrow mesenchymal stromal cells in myelodysplastic syndrome and multiple myeloma

**DOI:** 10.1111/cpr.12819

**Published:** 2020-05-05

**Authors:** Hayoung Choi, Yonggoo Kim, Dain Kang, Ahlm Kwon, Jiyeon Kim, Jung Min Kim, Sung‐Soo Park, Yoo‐Jin Kim, Chang‐Ki Min, Myungshin Kim

**Affiliations:** ^1^ Catholic Genetic Laboratory Center Seoul St. Mary's Hospital College of Medicine The Catholic University of Korea Seoul Korea; ^2^ Department of Biomedicine & Health Sciences Graduate School The Catholic University of Korea Seoul Korea; ^3^ Department of Laboratory Medicine College of Medicine The Catholic University of Korea Seoul Korea; ^4^ Heimbiotek, Inc. Gyeonggi‐do Korea; ^5^ Department of Hematology Leukemia Research Institute Seoul St. Mary's Hematology Hospital College of Medicine The Catholic University of Korea Seoul Korea

**Keywords:** *CDKN2A*, mesenchymal stromal cells, multiple myeloma, myelodysplastic syndrome, proliferation

## Abstract

**Objective:**

The objective of this study was to explore characteristics of bone marrow mesenchymal stromal cells (BM‐MSCs) derived from patients with myelodysplastic syndrome (MDS) and multiple myeloma (MM).

**Methods:**

BM‐MSCs were recovered from 17 of MDS patients, 23 of MM patients and 9 healthy donors and were passaged until proliferation stopped. General characteristics and gene expression profiles of MSCs were analysed. In vitro, ex vivo coculture, immunohistochemistry and knockdown experiments were performed to verify gene expression changes.

**Results:**

BM‐MSCs failed to culture in 35.0% of patients and 50.0% of recovered BM‐MSCs stopped to proliferate before passage 6. MDS‐ and MM‐MSCs shared characteristics including decreased osteogenesis, increased angiogenesis and senescence‐associated molecular pathways. In vitro and ex vivo experiments showed disease‐specific changes such as neurogenic tendency in MDS‐MSCs and cardiomyogenic tendency in MM‐MSCs. Although the age of normal control was younger than patients and telomere length was shorter in patient's BM‐MSCs, they were not different according to disease category nor degree of proliferation. Specifically, poorly proliferation BM‐MSCs showed *CDKN2A* overexpression and *CXCL12* downregulation. Immunohistochemistry of BM biopsy demonstrated that CDKN2A was intensely accumulation in perivascular BM‐MSCs failed to culture. Interestingly, patient's BM‐MSCs revealed improved proliferation activity after *CDKN2A* knockdown.

**Conclusion:**

These results collectively indicate that MDS‐MSCs and MM‐MSCs have common and different alterations at various degrees. Hence, it is necessary to evaluate their alteration status using representative markers such as CDKN2A expression.

## 1 INTRODUCTION

It has been well established that most haematologic malignancies are caused by genetic alterations in haematopoietic stem cells. Several genetic alterations including fusions and mutations are disease‐specific. Some mutations are more commonly detected in malignancies of specific lineage while others occur regardless of lineage. Another important aspect is that haematopoietic microenvironment is altered in haematologic malignancies. Bone marrow mesenchymal stromal cells (BM‐MSCs) are mainly responsible for the establishment of the haematopoietic microenvironment.^1^ Studies have shown that patient's BM‐MSCs do not harbour the same genetic abnormalities present in malignant cells. Common or disease‐specific genetic alteration in haematologic malignancies has not been identified.^2^, ^3^, ^4^, ^5^ BM‐MSCs from patients with myelodysplastic syndrome (MDS) and acute myeloid leukaemia (AML) exhibit a wide range of functional and molecular alterations.^4^, ^6^ These alterations are not restricted to malignancies in myeloid lineage. Ex vivo coculture model has indicated that acute lymphoblastic leukaemia cells can create a leukaemic niche in the direction of attracting leukaemic cells and repelling normal haematopoietic cells.^7^ BM‐MSCs from multiple myeloma (MM) patients also show much lower proliferative activity and different gene expression compared with normal BM‐MSCs.^8^ Therefore, BM‐MSCs are considered as active players in the pathophysiology of haematologic malignancies rather than passive by‐standers in haematopoietic microenvironment. They are even considered, as possible therapeutic targets.^8^, ^9^, ^10^ However, most of previous studies on individual haematologic malignancies compared characteristics of patient's BM‐MSCs to those of normal MSCs from healthy donors (normal MSCs). Little is known about whether the alteration has occurred via a common or distinct mechanism. In addition, it remains unclear whether there is a difference in the alteration by disease category or whether there are individual differences within the same disease.

In the present study, we derived BM‐MSCs from MDS (MDS‐MSCs) and MM patients (MM‐MSCs) and evaluated their characteristics through gene expression and functional analysis after their expansion ex vivo. In parallel, we conducted coculture experiment and analysed gene expression changes of normal MSCs after priming with MDS or MM cell lines. We also tried to explore common and different alterations in MDS‐MSCs and MM‐MSCs including their mechanisms. Moreover, we investigated whether the alteration varied individually even within the same disease.

## 2 MATERIALS AND METHODS

### 2.1 Ethics statement

This study was approved by the Institutional Review Board of Seoul St. Mary's hospital (KC12SISE0594 and KC11SISE0001). BM samples were collected after obtaining written informed consent from participants according to Helsinki Declaration and local regulations.

### 2.2 Culture and identification of MSCs from patient's BM

BM‐MSCs were recovered from BM samples of healthy donors (normal, n = 9) and patients with MDS (n = 17) or MM (n = 23). One millilitre of BM aspirates was mixed with 10 mL complement culture medium (CCM) containing α‐modified minimum essential medium (α‐MEM; Gibco, Grand Island, NY, USA), 15% foetal bovine serum (FBS, Gibco), 1% glutamax™‐I (Gibco) and 1% penicillin‐ streptomycin (Gibco). Cells were then cultured in a T‐25 culture flask at 37°C with 5% CO_2_. Cells were harvested at 7‐10 days using 0.25% (w/v) trypsin‐EDTA (Gibco) and cultured on 150 mm culture dishes (SPL Life Sciences, Pocheon‐si, Korea). Cultured BM‐MSCs were harvested at 70‐80% confluency and seeded 1 × 10^5^ cells on 150 mm culture dishes (SPL Life Sciences). Normal MSCs were kindly provided by Texas A&M Health Science Center, College of Medicine, Institute for Regenerative Medicine at Scott & White (Temple, TX, USA) (n = 4) and Catholic Institute of Cell Therapy (n = 5), College of Medicine, The Catholic University of Korea (Seoul, Korea), and expanded under the same conditions as patients' BM‐MSCs.

Immunophenotypes of cultured BM‐MSCs were analysed at passage 3 (P3) using mouse anti‐human monoclonal antibodies including CD105, CD90, CD73, CD34, CD45, CD11b, CD79a and HLA‐DR (BD Biosciences, CA, USA). A total of 1 × 10^5^ cells were applied to a FACSCalibur cytometer (BD Biosciences) and analysed using CellQuest™Proversion 6.0 software (BD Biosciences).

To calculate population doubling time (PDT) of each passage, cells were counted using a disposal hemocytometer C‐Chip (SystemBükerTürk; Incyto, Cheonan, Korea). Passaging was proceeded at a seeding concentration of 1 × 10^5^ cells per dish. The passaging was repeated until BM‐MSCs stopped proliferating. PDT was calculated using an algorithm available online (http://www.doubling-time.com/compute.php). We measured cellular fraction in apoptosis and necrosis at P3 using an Apoptosis/Necrosis Detection Kit (ab176749, Abcam, Cambridge, MA, USA) by flow cytometry (FACSCalibur, BD Biosciences) and CellQuest™ Proversion 6.0 software (BD Biosciences) according to manufacturer's instruction.

### 2.3 Gene expression profile by microarray analysis

For gene expression analysis, RNAs were extracted from three samples of normal MSCs, six samples of MDS‐MSCs, and six samples of MM‐MSCs at P3 using RNeasy^®^ Mini Kit (Qiagen, Hilden, Germany). We included patient's BM‐MSCs with various proliferative activities. Microarray analysis was carried out using SurePrint G3 Hmn GE 8Χ 60K V2 Microarray Kit (Agilent Technologies, Santa Clara, CA, USA). Microarray images were obtained using a High‐Resolution Microarray Scanner (Agilent Technologies). Expression levels were quantified using Feature Extraction software 10.7.3.1 (Agilent Technologies). All data normalization and the gene selection of 2‐fold changes were performed using GeneSpring GX 7.3 (Agilent Technologies). Functional annotation of differentially expressed genes (DEGs) was carried out using Gene Ontology (GO) Consortium (http://www.geneontology.org/index.shtml). Gene classification was based on searches executed with GeneCards (http://www.genecards.org/) and DAVID (http://david.abcc.ncifcrf.gov/).

### 2.4 Reverse transcription quantitative PCR (RT‐qPCR)

RT‐qPCR was carried out to measure gene expression including cyclin‐dependent kinase inhibitor 2A (*CDKN2A*) (HS00923894_m1), cyclin‐dependent kinase inhibitor 2B (*CDKN2B*) (Hs00793225_m1), C‐X‐C Motif Chemokine Ligand 12 (*CXCL12*) (Hs00171022_m1), toll‐like receptor 4 (*TLR4*) (Hs00152939_m1), nestin (*NES*) (Hs00707120_s1) and troponin T1 (*TNNT1*) (Hs00162848_m1). Experiments were performed using TaqMan^®^ gene expression assay and master mix (Applied Biosystems, Foster city, CA, USA) on an ABI 7500 Real‐Time PCR system (Applied Biosystems). Gene expression levels were estimated in triplicates using the 2^−ΔΔCt^ method with *GAPDH* (Hs99999905_m1) as internal control for normalization.

### 2.5 Telomere length analysis

We measured telomere length of BM‐MSCs according to our previous protocol.^11^ Telomere‐specific primers and the 36b4 primers were used. All PCRs were performed on the Rotor‐Gene Q real‐time instrument (Qiagen). The average telomere length in a cell was calculated as the telomere‐to‐single copy gene (T/S) ratio using Rotor‐Gene Q software 2.0.2.

### 2.6 In vitro oesteogenic, chondrogenic, adipogenic, neurogenic and cardiomyogenic differentiation

We seeded 1 × 10^5^ BM‐MSCs at P3 into each well of a 6‐well plate (Nunc, Shanghai, China). Culture medium was changed into differentiation medium when cells reached 70% confluency. After culturing for three weeks, mesodermal differentiation was analysed after special staining with the same procedure as described in our previous study.^12^ Briefly, adipogenic differentiation was induced using a StemPro™ Adipogenesis Differentiation Kit (Gibco, Grand Island, NY, USA) and observed after Oil Red‐O staining (Sigma‐Aldrich, St. Louis, MO, USA). Chondrogenic differentiation was performed using a StemPro™ Chondrogenesis Differentiation Kit (Gibco) and stained with 1% alcian blue solution (ScienCell, Carlsbad, CA, USA) and 0.1% nuclear fast red solution (ScienCell). Osteogenic differentiation was induced with a StemPro™ Osteogenesis Differentiation Kit (Gibco) and analysed after staining with 2% Alizarin Red Solution (ScienCell).

In addition, we tried to differentiate BM‐MSCs into neural cells and cardiomyocytes according to our previous protocol.^13^ The medium was replaced with Neural Induction Medium and supplement (Gibco). After two weeks, cells were stained with anti‐SOX2 (Abcam, Cambridge, MA, USA) and anti‐Nestin antibody (Abcam) and observed using a conformal system. Cardiomyocyte differentiation medium A (Gibco) was replaced when cells reached 70% confluency. After two days, cardiomyocyte differentiation medium B (Gibco) was applied for two days. Cells were then cultured in cardiomyocyte maintenance medium (Gibco) for two weeks. Differentiation was confirmed using a C2 + confocal system (Nikon, NY, USA) after staining with anti‐alpha actinin (Abcam) and anti‐cardiac troponin T antibody (Abcam).

### 2.7 Ex vivo coculture experiment

To determine whether normal MSCs underwent similar change to patient's BM‐MSCs after direct contact with malignant cells, we performed ex vivo coculture experiment. CD34 + haematopoietic stem cells (HSCs), SKM1 (MDS) and IM‐9(MM) cell lines were purchased from Gibco (StemPro^®^ 34 + Cell Kit), Japanese Collection of Research Bioresources Cell Bank (JCRB, Osaka, Japan) and Korea Cell Line Bank (KCLB, Seoul, Korea), respectively. We chose SKM1 cell line for MDS because true MDS cell line did not exist yet. SKM1 cell line was established from a patient with progression to myelomonocytic leukaemia in MDS; therefore, the cell line was good to understand the mechanism of disease progression but not really represent low‐risk MDS.^14^ Normal MSCs from four healthy donors (1 × 10^4^) were seeded into 100 mm plates and incubated at 37°C in CCM for 1 day. These MSCs were then cultured alone (unprimed) or cocultured (primed) with 2.5 × 10^4^ CD34 + HSCs, SKM1, or IM‐9 cell lines. Primed and unprimed MSCs were harvested after 10 days and subjected to RNA sequencing.

### 2.8 Analysis of gene expression changes by RNA‐sequencing

One gram of total RNA was processed to prepare mRNA sequencing library using a TruSeq stranded mRNA sample preparation kit (Illumina, San Diego, CA, USA) according to the manufacturer's instruction. Products were then purified and enriched with PCR to create the final cDNA library. Sequencing of the prepared library was conducted on a Nextseq system (Illumina) with 75 bp paired‐end reads. Mapping of high quality reads on Human reference genome (hg38), gene counting and differential analysis were performed using Strand NGS v.2.9 (Strand Genomics, CA, USA). For normalization of gene counting, DESeq algorithm was applied.^15^ To determine DEGs, cut‐off values for fold changes were log2 ratio ≥ 1 or ≤ −1, with raw read count ≥ 10. As biological replicates were not used in this study, cut‐off for statistical significance was not applied.

### 2.9 Mutation analysis by next‐generation sequencing (NGS)

We analysed and compared genetic mutations in patient's malignant cells and BM‐MSCs by NGS. DNAs were extracted from paired malignant cells and BM‐MSCs at P3 (n = 4) using Wizard^®^ Genomic DNA Purification kit (Promega, Madison, WI, USA). We used DNA Oncomine™ Comprehensive Panel v3M (Thermo Fisher Scientific, MA, USA) containing 134 genes according to the manufacturer's instruction. Elaborated sequence data in FASTQ format were adjusted and annotated according to hg19 human reference genome. NGS using clinical targeted panel (SM haematology NGS panel) containing 63 genes was performed to confirm the detected mutations.

### 2.10 CDKN2A, CD146 and CD271 immunohistochemistry (IHC) staining on BM biopsy

BM biopsy sections were prepared. After deparaffinization in xylene, sections were rehydrated in decreasing concentration of ethanol (100%, 95%, 80%) and subjected to IHC using Dako REAL™ EnVision™/HRP, Rabbit/Mouse (ENV) reagent of the kit (DAKO, Glostrup, Denmark). Antigen was retrieved using Tris‐EDTA buffer (pH 9.0, ab93684; Abcam) for 15 min at 95°C. These slides were then incubated in 3% hydrogen peroxide at room temperature for 10 min to block endogenous peroxidases followed by incubation with each primary rabbit monoclonal antibody against CDKN2A/P16INK4a (ab108349, Abcam, 1:200 dilution), CD146 (ab75769, Abcam, 1:250 dilution) and CD271 (ab52987, Abcam, 1:200 dilution) at room temperature for 150 min. Following this, all slides were washed with PBS and then incubated with secondary antibody conjugated with Streptavidin‐peroxidase reagent at room temperature for 60 min. Bound peroxidase was then visualized after reacting with 3,3’diaminobenzidine (DAB substrate). Counterstaining was performed with haematoxylin.

### 2.11 CDKN2A knockdown (KD) experiment and real‐time cell monitoring

Since the CDKN2A might be important in proliferation activity in patient's BM‐MSCs, we performed *CDKN2A* KD experiment and analysed proliferation activity using a real‐time cell monitoring system. *CDKN2A* KD was performed by small interfering RNA (siRNA) with specific sequence of 5′‐CCGUAAAUGUCCAUUUAUATTUAUAAAUGGACAUUUAUGGTT‐3′ designed and synthesized by GenePharma (Shanghai GenePharma, Shanghai, China). BM‐MSCs at third passage (1.0 × 10^4^) were suspended in 150 μl of antibiotic‐free basic cell culture medium and seeded into each well of the E‐plate 16 (ACEA Biosciences Inc, San Diego, USA) and then installed into xCELLigence RTCA DP system (ACEA Biosciences Inc). After 24 hr, cells were transfected with 50 nM CDKN2A‐siRNA using Lipofectamine RNAiMAX (Invitrogen Life Technologies, Carlsbad, CA, USA). Untreated, Lipofectamine RNAiMAX treated and negative control siRNA (no genetic homology with human siRNA, GenePharma) treated cells were used as controls. The proliferation activity was measured every 10 min for following 3 days and analyses using RTCA software 2.0 (ACEA Biosciences Inc).

### 2.12 Data analysis

Data are presented as mean ± standard deviation (SD) of at least three independent determinations. Statistical differences between groups were determined using Student's t test or one‐way analysis of variance (ANOVA) followed by Bonferroni's post hoc test for multiple comparisons. Pearson's chi‐squared test and Pearson's correlation was utilized to analyse the relationship between analytic data. All analyses were performed using IBM^®^ SPSS^®^, version 24.0 (IBM Corp., Armonk, NY, USA). Differences were considered statistically significant at *P* < .05.

## 3 RESULTS

### 3.1 Impaired proliferation activity and differentiation potential of patient's BM‐MSCs

**Figure 1:**
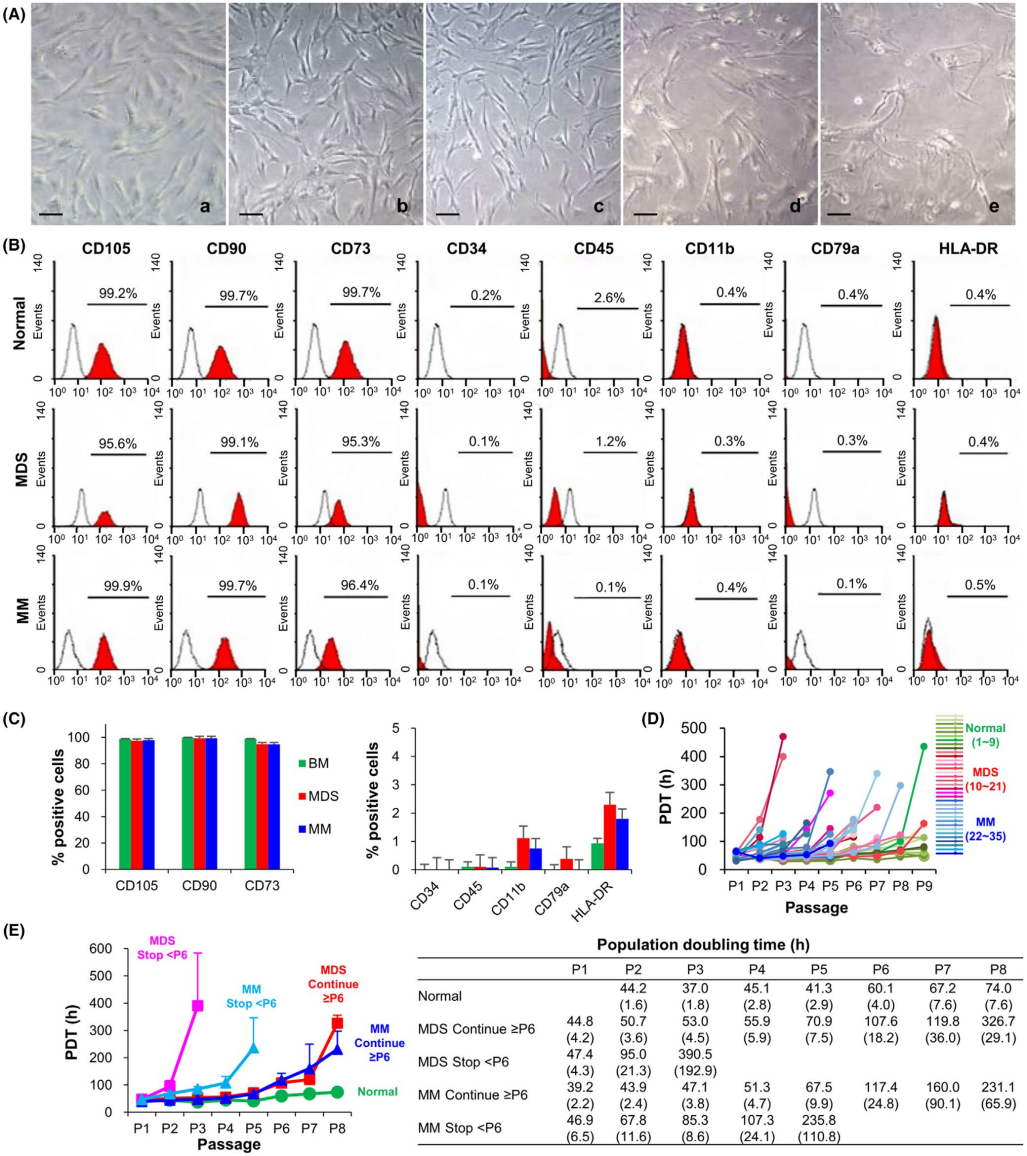
Characteristics of bone marrow mesenchymal stromal cells (BM‐MSCs) derived from patients with myelodysplastic syndrome (MDS) and multiple myeloma (MM). (A) Representative field of cultured BM‐MSCs at passage 3 (P3). From the left, normal BM‐MSCs (a), MDS‐MSCs (b) and MM‐MSCs (c) continued to proliferate after P6 (continue ≥ P6) while MDS‐MSCs (d) and MM‐MSCs (e) that stopped to proliferate before P6 (stop < P6) show more flattened cells. Scale bars, 100 μm. (B) Representative surface immunophenotype analysis of normal BM‐MSCs, MDS‐MSCs and MM‐MSCs at P3 using flow cytometry. Red histograms indicate surface marker staining. Black histograms represent isotype‐matched IgG controls. (C) MDS‐MSCs and MM‐MSCs show similar surface antigen to normal BM‐MSCs. (D) Growth kinetics of 35 BM‐MSCs through passages. Individual long‐term growth curves of normal BM‐MSCs (n = 9, green), MDS‐MSCs (n = 12, red) and MM‐MSCs (n = 14, blue). Population doubling time (PDT) is calculated based on the ratio of cells seeded versus cells harvested per passage. (E) Comparison among BM‐MSCs showing different proliferating activity. The results are presented as mean (SEM)

MSCs were successfully isolated and cultured from all BM samples from healthy donors. However, we did not observe BM‐MSC proliferation from 14 patients' samples (35.0%), including 5 of 17 (29.4%) patients with MDS and 9 of 23 (39.1%) patients with MM (Table 1). As showed in (Figure 1A), normal BM‐MSCs displayed a characteristic fibroblast‐like appearance, whereas some patient's BM‐MSCs were generally larger and more flat‐shaped epithelioid cells compared with normal controls. MSCs isolated from healthy donors or patients revealed typical immunophenotype of MSCs (positive for CD73, CD90 and CD105, but negative for HLA‐DR, CD11b, CD79a, CD34 and CD45) (Figure 1B,C). The proliferation activity of patients' BM‐MSCs was lower than that of normal controls, showing higher PDT from P3 (normal MSCs 38.0 ± 5.5 hr vs. MDS‐MSCs 193.6 ± 350.6 hr, *P* = .001 or vs. MM‐MSCs 67.7 ± 25.9 hr, *P* < .001). Patients' BM‐MSCs revealed different degree of proliferation activities. Half of them stopped to proliferate before P6 (total: 13/26, 50.0%, MDS‐MSCs 5/12, 41.7%; MM‐MSCs 8/14, 57.1%) (Figure 1D,E).

**Table 1:** Clinical and laboratory characteristics of patients included in the study at diagnosis

ID	Age	Sex	Diagnosis	MC (%)	Karyotype of MC	MSC P
MDS1	49	F	MDS‐EB1	6	47,XX,+8	7
MDS2	70	M	MDS‐EB1	8	46,XY	6
MDS3	78	F	MDS‐U	1	46,XX	8
MDS4	70	M	MDS‐MLD	1	46,XY	8
MDS5	33	F	MDS‐MLD	1	46,XX	10
MDS6	74	F	MDS‐EB2	18	46~48,XX,‐5,+8,add(14)(p11.2),+15,‐17,‐22,+1~2mar[cp17]/46,XX[3]	6
MDS7	75	M	MDS‐EB2	18	46,XY	7
MDS8	81	M	MDS‐EB1	8	46,XY	3
MDS9	72	F	MDS‐EB2	18	49~51,XX,t(1;1)(p13;p36.1),+2,del(11)(q21q23),?t(16;21)(q22;q22),+r,+2~3mar[cp6]/46,XX[14]	3
MDS10	19	M	MDS‐SLD	1	47,XY,+8	3
MDS11	56	M	MDS‐EB2	13	46,XY	5
MDS12	50	M	MDS‐MLD	3	46,XY,del(12)(p12)[20]	5
MDS13	43	M	MDS‐U	1	No mitotic cell	0
MDS14	65	M	MDS‐EB2	12	45~46,XY,del(?3)(p21),der(5)t(3;5)(p14;q14),‐17,der(22)t(11;22)(q13;p11.2),+mar1,+mar2[cp10]/45~46,XY,‐3,der(5)t(3;5),del(7)(q11.2q32),‐17,add(20)(q13.1),+mar1,+mar2[cp8]/ 46,XY[2]	0
MDS15	56	F	MDS‐EB2	11	46,XX,dup(1)(q21q32)[3]/ 46,XX[17]	0
MDS16	37	M	MDS‐EB2	11	47,XY,+8[20]	0
MDS17	41	M	RCMD	4	48,XY,+8,+9[3]/ 46,XY[17]	0
MM1	77	M	MM IgG(κ)	80	46,X,Y,der(3)t(1;3)(q12;p26),add(6)(q27),add(19)(p13.3), +mar[cp6]/ 46,XY[14]	9
MM2	58	M	MM IgA(λ)	4	46,XY[20]	6
MM3	56	M	MM IgG(λ)	67	46,XY,inv(9)(p12q13)[30]	8
MM4	64	F	LCD (λ)	60	46,XX[20]	6
MM5	63	M	MM IgG(λ)	80	56,XY,+1,der(1;6)(q10;p10),add(2)(q22),+del(4)(q21),+5,+9,+add(11)(q23),add(14)(q32),+15,+18,+19,+19,+21[2]/ 46,XY[18]	7
MM6	48	M	MM IgG(λ)	7	46,XY[20]	5
MM7	81	F	MM IgA(κ)	56	46,XX[20]	5
MM8	62	F	MM IgA(λ)	90	42,X,‐X,,‐13,‐14,add(15)(p13),‐16,inc[cp3]/46,XX[17]	3
MM9	57	F	MM IgA(λ)	26	47~48,XX,+add(1)(p13)X2,t(4;8)(p16;p21),der(6)t(6;6)(q13;p11.2),+7,+9,‐12,add(13)(p13),der(14;15)(q10;q10)[cp4]/ 46,XX[16]	2
MM10	69	M	MM IgG(λ)	40	53~55,XY,+3,+5,+7,+9,add(10)(q22),add(14)(q24),+15,add(16)(p13.3),+19,der(20)t(1;20)(q21;q13.3),+21[cp2]/46,XY[18]	3
MM11	56	M	LCD (κ)	90	46,XY[20]	4
MM12	76	F	MM IgA(κ)	31	42,X,‐X,i(1)(q10),i(5)(p10),i(9)(q10),‐13,‐14,‐16,add(22)(q13)[21]/46,XX[9]	4
MM13	70	M	MM IgA(κ)	80	43,XY,dup(1)(q12q44),t(8;13)(q24.1;q12),‐13,‐14,‐20[12]/86,idemx2[2]/ 46,XY[6]	3
MM14	70	M	MM IgG(κ)	30	54,XX,+5,+7,+9,+14,+15,+16,+21,+21,inc[1]/46,XY[19]	5
MM15	53	F	LPL IgM(λ)	95	47,XX,+12,i(18)(q10)[3]/48,XX,+3,del(15)(q11.2q15),+18,i(18)(q10)x2[4]/46,XX[23]	0
MM16	71	M	LCD (κ)	80	46,XY[20]	0
MM17	63	M	LCD (κ)	65	46,XY,+1,der(1;13)(q10;q10),t(2;19)(p21;p13.1),der(14)t(11;14)(q13;q32)[5]/46,XY[15]	0
MM18	54	F	MM IgG(κ)	95	46,XX[30]	0
MM19	80	F	MM IgG(κ)	95	48,XX,del(1)(p13),+del(1)(p13)x2,‐4,der(4)t(1;4)(p32;p16),t(7;14)(q32;q32),+11,‐13,add(18)(q23),+19[7]/48,X,‐X,+del(1)(p13)x2,t(2;8)(p11.2;q24.1),add(3)(q25),del(4)(p14),+6,der(6;8)(p10;q10),+9,+11,‐13[2]/ 46,XX[11]	0
MM20	46	M	MM IgG(κ)	70	46,XY[10]	0
MM21	65	M	MM IgA(λ)	60	46,XY[20]	0
MM22	64	F	MM IgG(λ)	80	46,XX[20]	0
MM23	65	F	MM IgA(κ)	99	46,XX[20]	0

Abbreviations: ID, identification; MC, malignant cell; MSC P, Possible subculture passage of bone marrow mesenchymal stromal cells; MDS‐EB, myelodysplastic syndrome with excess blasts; MDS‐U, MDS, unclassifiable; MLD, multilineage dysplasia; SLD, single lineage dysplasia; RCMD, refractory cytopenia with multilineage dysplasia; MM, multiple myeloma; LCD, light chain disease; LPL, lymphoplasmacytic lymphoma.

The donor age was inversely correlated with T/S ratio (*R* = −0.587, *P* < .001) and *CXCL12* expression (*R* = −0.493, *P* = .004) (Figure S1A). Although the age of normal control was younger than patients (*P* < .001), it was not different between disease category nor among MSCs continued to proliferate after P6, stopped to proliferate before P6, and culture failure in patients (Figure S1B). In addition, T/S ratio was lower in patients than normal control (*P* = .001), but it was not different according to disease category nor degree of proliferation (Figure S1C). *CXCL12*, a gene required for haematopoietic stem‐cell maintenance^16^ expression was also significantly lower in patients than normal control (*P* = .004), but it was not different between disease category in patients (Figure S1D). Then, we categorized disease state according to revised international prognostic system (IPSS‐R) and international staging system (ISS) for MDS and MM, respectively. Patients were grouped as high‐risk (IPSS‐R high/very high and ISS stage II/III) and low‐risk (IPSS‐R very low/low/intermediate and ISS stage I). Interestingly, there was significant difference of proliferation activity between two risk groups (*P* = .015) (Table S1).

Flow cytometry revealed that patient's BM‐MSCs showed higher apoptotic and/or necrotic fraction than normal MSCs (7.6% ± 2.1% vs. 2.7% ± 0.9%, *P* = .036) (Figure S2). In the aspect of differentiation, osteogenic potential was decreased in patient's BM‐MSCs and adipogenic potential was decreased in MM‐MSCs. Chondrogenic potential of patients’ BM‐MSCs was similar to that of normal BM‐MSCs (Figure S3).

### 3.2 Altered gene expression profile in patient's BM‐MSCs compared to normal BM‐MSCs

**Figure 2:**
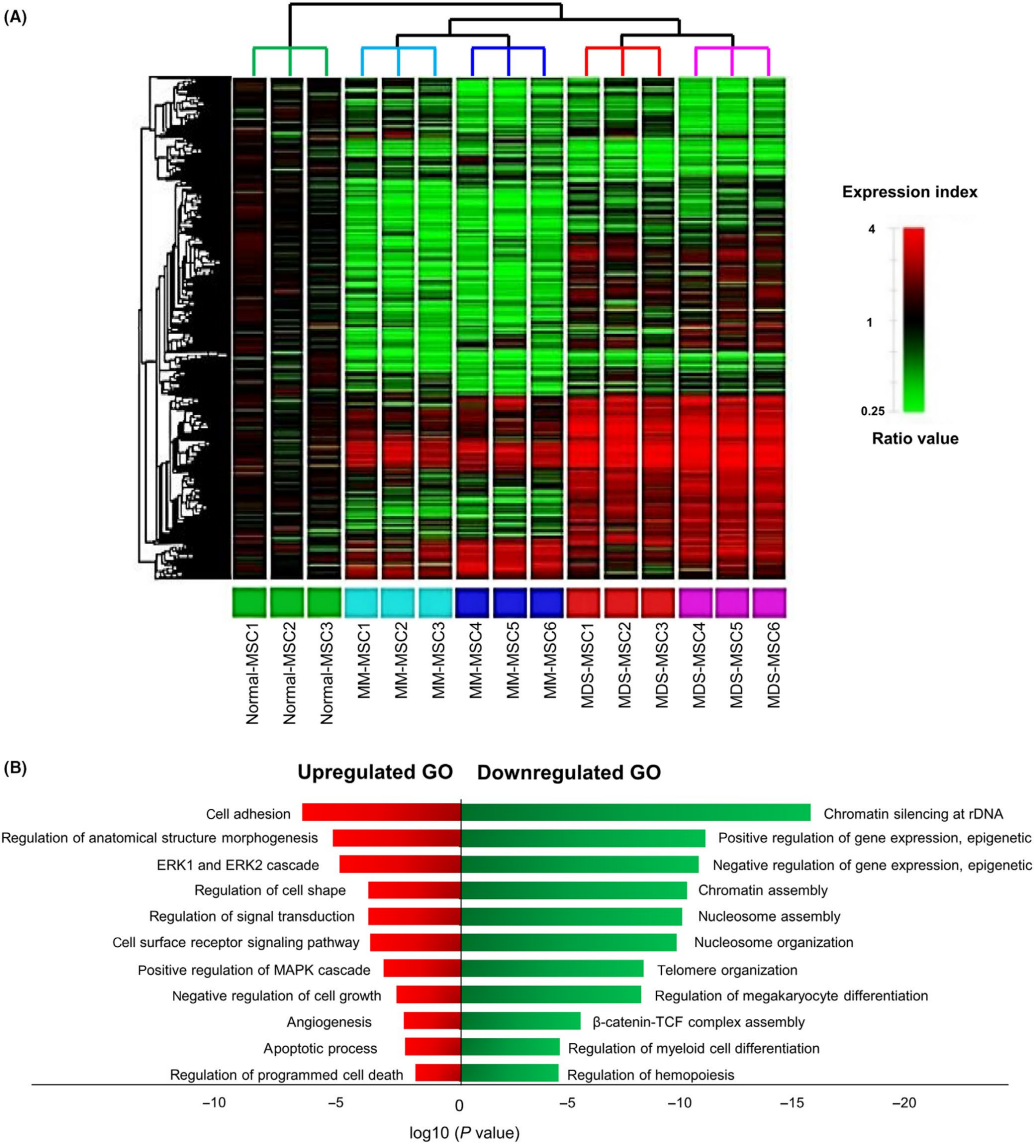
Gene expression profiles in bone marrow mesenchymal stromal cells (BM‐MSCs) derived from normal (n = 3) and patients with myelodysplastic syndrome (MDS, n = 6) and multiple myeloma (MM, n = 6). (A) Heat map showing differentially expressed genes relative to normal BM‐MSCs. Red spots indicate upregulated transcripts. Green spots indicate downregulated transcripts. The dendrogram is derived by unsupervised hierarchical clustering of gene expression distinguishing MDS‐MSCs from MM‐MSCs. Further clustering showed distinct groupings between BM‐MSCs that continued to proliferate after passage 6 (P6) and those that stopped to proliferate before P6. (B) General functional classification of common genes with expression level changed more than 2‐fold in MDS‐MSCs and MM‐MSCs compared to normal BM‐MSCs. Gene Ontology (GO) analysis within target genes of significantly altered transcripts was performed using the database for annotation, visualization and integrated discovery (DAVID) bioinformatics tool. Enriched GO biological processes were identified and listed according to their enrichment *P* value (*P* < .05) and False discovery rate (FDR < 0.25). Both *P* and FDR values were obtained using DAVID 2.1 statistical function classification tool

Hierarchical cluster analysis^17^ revealed that gene expression profile firstly distinguished patients' BM‐MSCs from normal BM‐MSCs. It then divided again by disease category (Figure 2A). In patient's BM‐MSCs, genes associated with “biological processes including cell adhesion,” “ERK1 and ERK2 cascade,” “regulation of cell shape,” “positive regulation of MAPK cascade,” “negative regulation of cell growth,” “apoptotic process,” “regulation of programmed cell death” and “vascular development” were significantly upregulated. On the other hand, genes associated with “chromatin silencing at rDNA,” “epigenetic regulation of gene expression,” “chromatin assembly,” “nucleosome assembly,” “nucleosome organization,” “regulation of megakaryocyte differentiation,” “regulation of myeloid cell differentiation,” “regulation of hemopoiesis” and “β‐catenin‐TCF complex assembly” were significantly downregulated (Figure 2B).

**Figure 3:**
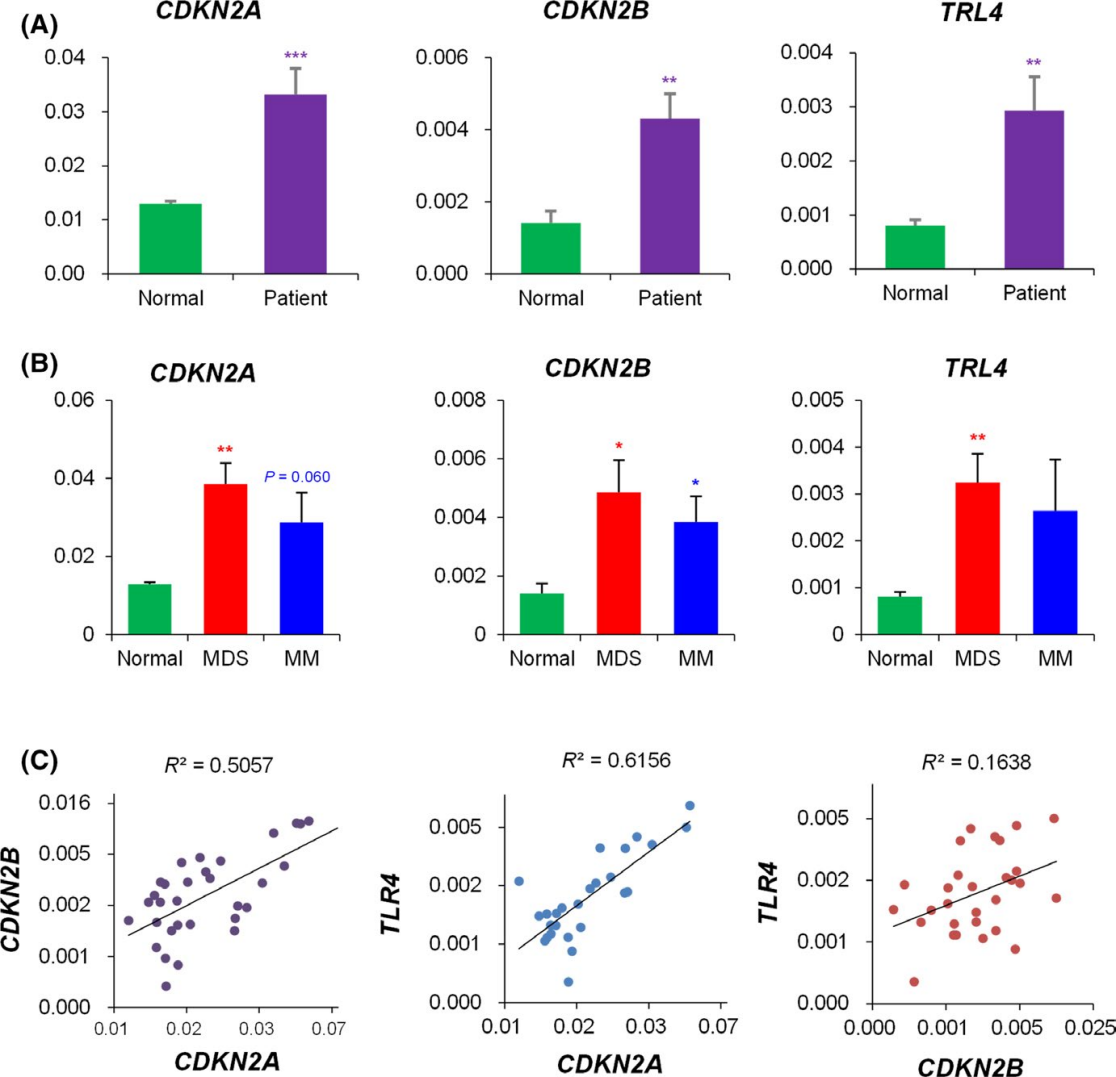
Expression levels of CDKN2A, CDKN2B and TLR4 genes and their correlations. (A) Comparison of gene expression between normal control and patient's bone marrow mesenchymal stromal cells (BM‐MSCs), (B) Comparison of gene expression of BM‐MSCs from normal controls, myelodysplastic syndrome (MDS) and multiple myeloma (MM) patients, quantified by RT‐qPCR. Experiments were performed in technical triplicates. Results are presented as mean ± SEM calculated from data obtained from independent samples of normal (n = 9), MDS (n = 12) and MM (n = 14). GAPDH gene was used as an endogenous control. (*P < .05, **P < .01, ***P < .001). (C) Correlation between CDKN2A, CDKN2B and TLR4 gene expression

*CDKN2A* and *CDKN2B* expression was significantly higher in patients’ BM‐MSCs compared with those in normal BM‐MSCs. *TLR4* gene belonging to the family of Toll‐like receptors^18^ was highly expressed in patients’ BM‐MSCs compare with that in normal BM‐MSCs (Figure 3A,B). *CDKN2A* expression had a positive correlation with *CDKN2B* expression (*P* < .001). Expression levels of *CDKN2A* and *CDKN2B* were positively correlated with *TLR4* expression level (*P* < .001 and *P* < .001, respectively) (Figure 3C).

### 3.3 Different gene expression and functional alterations in patient's BM‐MSCs

By disease category, genes associated with “cell morphogenesis” and “neuron development” were highly expressed in MDS‐MSCs while genes associated with “heart development” and “receptor‐mediated endocytosis” were expressed highly in MM‐MSCs. Genes associated with “translational elongation” including ribosomal protein small and large subunits were downregulated in MM‐MSCs (Table 2).

**Table 2:** Different gene expression in patient's bone marrow mesenchymal stromal cells (MSCs)

	*P* value	FDR
Myelodysplastic syndrome (MDS)‐MSC 2‐fold up GO		
Cell morphogenesis	8.59 × 10^−4^	1.32 × 10^−2^
Neuron development	1.91 × 10^−3^	2.91 × 10^−2^
Cell projection morphogenesis	2.15 × 10^−3^	3.28 × 10^−2^
Cell part morphogenesis	2.89 × 10^−3^	4.38 × 10^−2^
Neuron projection morphogenesis	3.29 × 10^−3^	4.98 × 10^−2^
Regulation of cell shape	3.45 × 10^−3^	5.21 × 10^−2^
Neurogenesis	4.00 × 10^−3^	6.01 × 10^−2^
Generation of neurons	5.23 × 10^−3^	7.80 × 10^−2^
Axonogenesis	7.08 × 10^−3^	1.04 × 10^−1^
Cell morphogenesis involved in differentiation	7.38 × 10^−3^	1.08 × 10^−1^
Cell morphogenesis involved in neuron differentiation	1.07 × 10^−2^	1.54 × 10^−1^
Regulation of cell morphogenesis	1.79 × 10^−2^	2.44 × 10^−1^
Protein hetero‐oligomerization	2.21 × 10^−2^	2.92 × 10^−1^
Response to oestrogen stimulus	3.33 × 10^−2^	4.08 × 10^−1^
Regulation of cell‐matrix adhesion	3.85 × 10^−2^	4.56 × 10^−1^
Regulation of protein transport	4.30 × 10^−2^	4.94 × 10^−1^
Monocyte chemotaxis	4.54 × 10^−2^	5.13 × 10^−1^
Apoptotic mitochondrial changes	4.95 × 10^−2^	5.45 × 10^−1^
MDS‐MSC 2‐fold down GO		
DNA metabolic process	3.07 × 10^−6^	4.76 × 10^−3^
DNA repair	8.11 × 10^−5^	1.19 × 10^−1^
Haemopoietic or lymphoid organ development	1.19 × 10^−4^	1.70 × 10^−1^
Regulation of B‐cell differentiation	2.85 × 10^−4^	3.62 × 10^−1^
Regulation of myeloid cell differentiation	2.93 × 10^−4^	3.71 × 10^−1^
Multiple myeloma (MM)‐MSC 2‐fold up GO		
Heart development	1.78 × 10^−4^	2.82 × 10^−3^
Receptor‐mediated endocytosis	2.87 × 10^−3^	4.45 × 10^−2^
Response to oestrogen stimulus	3.29 × 10^−3^	5.09 × 10^−2^
Muscle organ development	5.80 × 10^−3^	8.81 × 10^−2^
Protein‐DNA complex assembly	6.72 × 10^−3^	1.01 × 10^−1^
Nucleosome organization	7.45 × 10^−3^	1.12 × 10^−1^
Regulation of synaptic transmission	1.31 × 10^−2^	1.89 × 10^−1^
Regulation of protein kinase cascade	1.74 × 10^−2^	2.43 × 10^−1^
Striated muscle tissue development	2.29 × 10^−2^	3.08 × 10^−1^
Decidualization	2.32 × 10^−2^	3.10 × 10^−1^
Dephosphorylation	2.42 × 10^−2^	3.22 × 10^−1^
Muscle tissue development	2.83 × 10^−2^	3.66 × 10^−1^
Chromatin assembly or disassembly	3.03 × 10^−2^	3.86 × 10^−1^
Maternal placenta development	3.04 × 10^−2^	3.88 × 10^−1^
Regulation of hormone secretion	3.35 × 10^−2^	4.18 × 10^−1^
Regulation of secretion	3.37 × 10^−2^	4.20 × 10^−1^
Cellular protein complex disassembly	3.85 × 10^−2^	4.63 × 10^−1^
Vasculogenesis	3.96 × 10^−2^	4.73 × 10^−1^
MM‐MSC 2‐fold down GO		
Translational elongation	6.11 × 10^−6^	9.08 × 10^−5^
Translation	3.83 × 10^−3^	5.54 × 10^−2^
mRNA metabolic process	2.20 × 10^−2^	2.82 × 10^−1^
RNA splicing	3.13 × 10^−2^	3.77 × 10^−1^
Tissue homeostasis	4.80 × 10^−2^	5.18 × 10^−1^

Note

The enriched Gene Ontology (GO) biological processes were identified and listed according to their enrichment *P* value (<0.05) and False discovery rate (FDR).

We carried out further in vitro experiments to differentiate patients’ BM‐MSCs into neural cells and cardiomyocytes. Interestingly, MDS‐MSCs and MM‐MSCs showed increased tendency for neurogenic and cardiomyogenic differentiation, respectively. During neurogenic differentiation, cells with retracted cytoplasm and cytoplasmic processes were more frequently observed in MDS‐MSCs. (Figure S4A). RT‐qPCR was performed against *NES* that showed increased expression in differentiated MDS‐MSCs (Figure S4C). Immunofluorescent staining showed higher expression levels of Nestin and Sox2 in neuron‐like cells derived from MDS‐MSCs compared with those from normal or MM‐MSCs (Figure S5A). During cardiomyogenic differentiation, elongated cells with perinuclear granular contents were more frequently observed in MM‐MSCs (Figure S4B). RT‐qPCR showed increased expression of *TNNT1* in differentiated MM‐MSCs (Figure S4D). Immunofluorescent staining of cardiac markers including α‐actinin and cardiac troponin T revealed strong positivity in cardiomyocyte‐like cells derived from MM‐MSCs compared to those from normal or MDS‐MSCs (Figure S5B).

### 3.4 Gene expression alteration of normal BM‐MSC after coculture with MDS and MM cell lines

Ex vivo coculture experiment showed that the gene expression profile of normal BM‐MSCs was changed after coculture with MDS or MM cell lines (Table 3). In SKM1‐primed BM‐MSCs, genes associated with “acute‐phase response,” “positive regulation of angiogenesis” and “neuromuscular process controlling posture” were upregulated. In IM‐9‐primed BM‐MSC, genes associated with “cell adhesion,” “positive regulation of ERK1 and ERK2 cascade,” “apoptotic process,” “programmed necrotic cell death,” “|MAPK cascade,” “angiogenesis” and “heart development” were upregulated while genes associated with “ossification,” “osteoblast differentiation” and “positive regulation of Wnt signalling pathway” were downregulated.

**Table 3:** Gene expression alteration of normal BM‐MSCs after coculture with SKM1 or IM‐9 cell line

	*P* value	FDR
SKM1‐primed BM‐MSCs 2‐fold up GO		
Acute‐phase response	1.12 × 10^−2^	1.49 × 10^−1^
Positive regulation of angiogenesis	1.18 × 10^−2^	1.56 × 10^−1^
Neuromuscular process controlling posture	4.04 × 10^−2^	4.45 × 10^−1^
Response to toxic substance	4.78 × 10^−2^	5.03 × 10^−1^
Uterus development	5.60 × 10^−2^	5.62 × 10^−1^
Platelet activation	8.11 × 10^−2^	7.02 × 10^−1^
Intracellular oestrogen receptor signalling pathway	8.29 × 10^−2^	7.10 × 10^−1^
Positive regulation of nitric‐oxide synthase activity	8.67 × 10^−2^	7.26 × 10^−1^
IM‐9‐primed BM‐MSCs 2‐fold up GO		
Cell adhesion	6.00 × 10^−6^	1.07 × 10^−4^
Positive regulation of ERK1 and ERK2 cascade	1.04 × 10^−4^	1.85 × 10^−3^
Apoptotic process	1.71 × 10^−4^	3.05 × 10^−3^
MAPK cascade	1.33 × 10^−3^	2.33 × 10^−2^
Programmed necrotic cell death	5.45 × 10^−3^	9.27 × 10^−2^
Angiogenesis	9.39 × 10^−3^	1.55 × 10^−1^
Heart development	2.64 × 10^−2^	3.79 × 10^−1^
Cardiocyte differentiation	6.06 × 10^−2^	6.72 × 10^−1^
Oncogene‐induced cell senescence	6.06 × 10^−2^	6.72 × 10^−1^
IM‐9‐primed BM‐MSCs 2‐fold down GO		
Ossification	9.88 × 10^−4^	1.66 × 10^−2^
Positive regulation of Wnt signalling pathway	5.26 × 10^−3^	8.55 × 10^−2^
Osteoblast differentiation	1.67 × 10^−2^	2.49 × 10^−1^
Positive regulation of osteoblast differentiation	3.02 × 10^−2^	4.06 × 10^−1^
Keratinocyte differentiation	6.25 × 10^−2^	6.66 × 10^−1^

Abbreviations: SKM1, myelodysplastic syndrome cell line; IM‐9, multiple myeloma cell line; bone marrow mesenchymal stromal cells (BM‐MSC); GO, Gene Ontology; FDR, False discovery rate.

### 3.5 Mutations mutually exclusive between patient's BM‐MSCs and malignant cells

Patient's malignant cells showed one or two disease‐associated mutations (Table 4). However, their BM‐MSCs did not harbour the same genetic mutations. We detected an *NF1* mutation c.8087C>T (p.Pro2696Leu) in MDS‐MSCs (MDS10). To determine whether the mutation was true and mutually exclusive between BM‐MSCs and malignant cells, repetitive NGS was performed using the same sample together with the patient's malignant cells and MDS‐MSCs at earlier passage (P2). Results showed that this *NF1* mutation had already existed in MDS‐MSCs from P2 with 2.0% of mutant allele fraction (MAF). It increased to 5.4% at P3. In addition, this mutation was not detected in patient's malignant cells after reanalysis using other targeted panel.

**Table 4:** Detected mutations in malignant cells and bone marrow mesenchymal stromal cells from four patients

ID	Cell type	Gene	DNA	Protein	Accession No.	Coverage	MAF
MDS5	MC	*MTOR*	c.5662T>G	p.Phe1888Val	COSM893814	2000	6.9%
	BM‐MSC (P3)	Not detected					
MDS10	MC	*U2AF1*	c.101C>A	p.Ser34Tyr	rs371769427 COSM1190367	2000	39.1%
	BM‐MSC (P3)	*NF1*	c.8087C>T	p.Pro2696Leu		1545	5.4%
	BM‐MSC (P2)	*NF1*	c.8087C>T	p.Pro2696Leu		1388	2.0%
MM3	MML_HSC	*BRAF*	c.1799T>A	p.Val600Glu	rs121913227 COSM476	1827	12.2%
	BM‐MSC (P3)	Not detected					
MM8	MC	*ATM*	c.3613C>T	p.Arg1205Cys	rs760928285 COSM6919288	1999	6.5%
	MC	*TP53*	c.817C>T	p.Arg273Cys	rs121913343 COSM10659	1959	5.9%
	BM‐MSC (P3)	Not detected					

Abbreviations: ID, identification; MDS, myelodysplastic syndrome; MM multiple myeloma; MC, malignant cell; BM‐MSC, bone marrow mesenchymal stromal cell; P, passage; MAF, mutant allele frequency.

### 3.6 Correlation of CDKN2A expression and impaired proliferation activity in patient's BM‐MSCs

**Figure 4:**
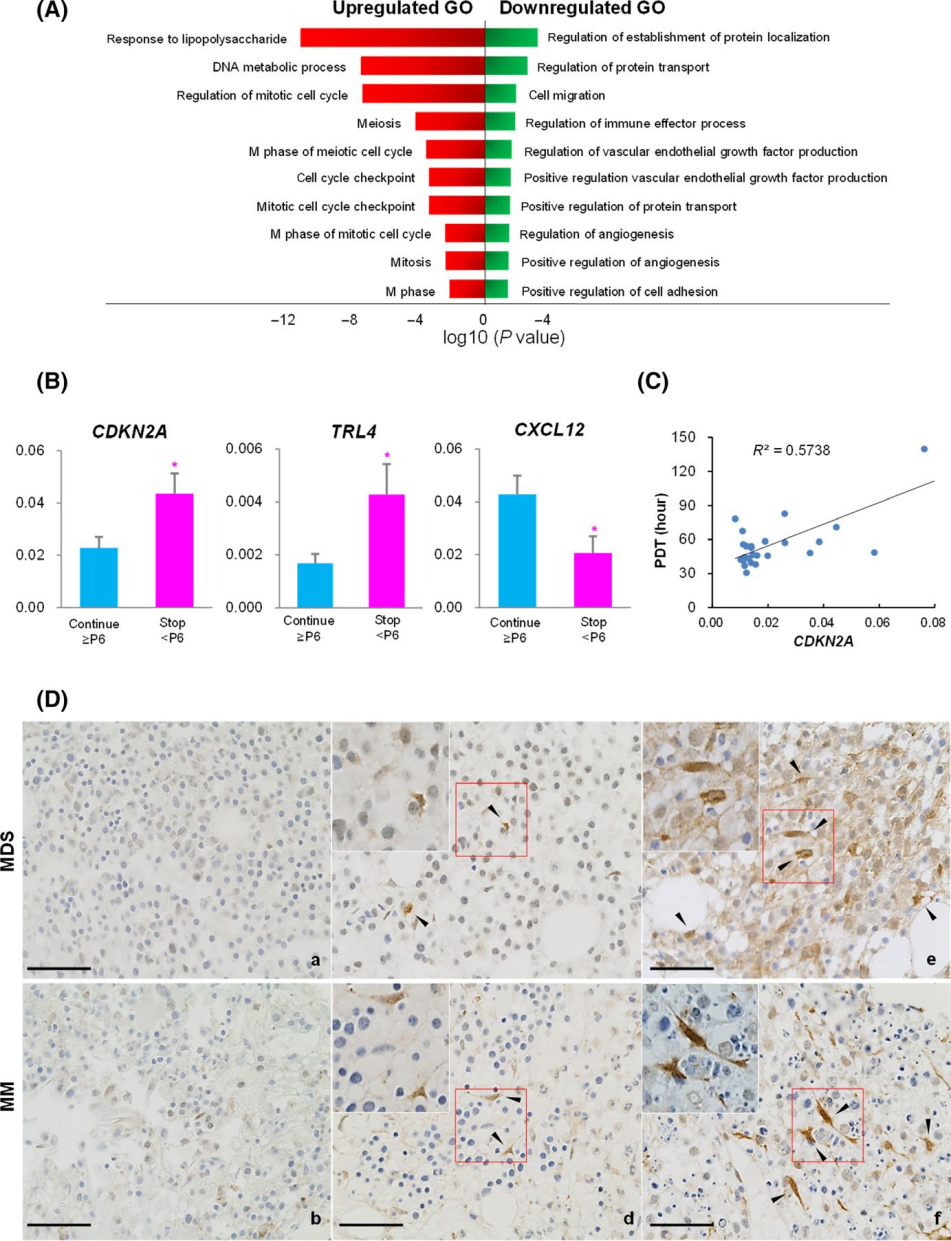
(A) Enriched Gene Ontology (GO) of genes upregulated and downregulated in patient's bone marrow mesenchymal stromal cells (BM‐MSCs) that continued to proliferate after passage 6 (Continue ≥ P6) compared to those that stopped to proliferate before P6 (Stop < P6). (B) Comparison of CDKN2A, TLR4, and CXCL12 expression in patient's BM‐MSCs between Continue ≥ P6 and Stop < P6 groups quantified by RT‐qPCR. Experiments were performed in technical triplicates. Results are presented as mean ± SEM calculated from data obtained from independent samples of patients with myelodysplastic syndrome (MDS, n = 12) or multiple myeloma (MM, n = 14). GAPDH gene was used as an endogenous control (*P < .05). (C) Correlation between CDKN2A expression and population doubling time (PDT) at P4. (D) Immunohistochemical staining of CDKN2A (stained brown) in BM biopsy sections from MDS and MM patients. Arrow heads indicate CDKN2A immunoreactive cells. Upper left square of each figure is magnification of red box. (a, b) Continue ≥ P6, (c, d) Stop < P6 and (e, f) Failure in colony formation. Images were obtained with a Carl Zeiss Microscopy GmbH (Carl Zeiss, Jena, Germany) equipped with a ProgRes MF camera (JENOPTIK, Jena, Germany). Original magnification, 400×. Scale bars, 100 μm

Hierarchical cluster analysis of microarray data indicated that patient's BM‐MSCs in each disease category were divided into two groups: (a) continue to proliferate after P6; and (b) stop to proliferate before P6. In the first group, genes functioning in “regulation of mitotic cell cycle,” “meiosis,” “M phase meiotic cell cycle” and “cell cycle checkpoint” were upregulated. And genes functioning in “cell migration,” “regulation of vascular endothelial growth factor production,” “regulation of angiogenesis” and “positive regulation of cell adhesion” were downregulated (Figure 4A). It was noteworthy that these differently gene expressing groups were in line with proliferation activity. *CDKN2A* and *TLR4* were highly expressed in the first group compared with those in the second group (*P* = .029 and *P* = .037, respectively). *CXCL12* expression was significantly low in the second group (*P* = .029) (Figure 4B). Interestingly, the value of *CDKN2A* expression showed a positive correlation with PDT of BM‐MSCs at P4 (*P* < .001) (Figure 4C).

Having observed higher *CDKN2A* expression in BM‐MSCs that stopped to proliferate before P6, we postulated that the value of *CDKN2A* expression might be correlated with the degree of impaired proliferation activity. To test this hypothesis, we implemented two of experiment. First, we determined *CDKN2A* expression levels in patients whose BM‐MSCs were not obtained because of culture failure. *CDKN2A* IHC was performed for patient's BM biopsy sample, because it was possible to observe the location and shape of immunoreactive cells (Figure 4D). CDKN2A was not or faintly stained in patient's BM whose MSCs continued to proliferate after P6. However, it showed slight to moderate cytoplasmic and nuclear staining on perivascular cells in BM whose MSCs stopped to proliferate before P6. Of note, we could observe intense CDKN2A accumulation in perivascular cells of BM that showed culture failure (Figure S6). The CDKN2A immunoreactive cells were thought to be BM‐MSC because they located on same locus as CD271 and/or CD146 immunoreactive cells (Figure S7). These results together indicated that the degree of impaired proliferation activity of patient's BM.

**Figure 5:**
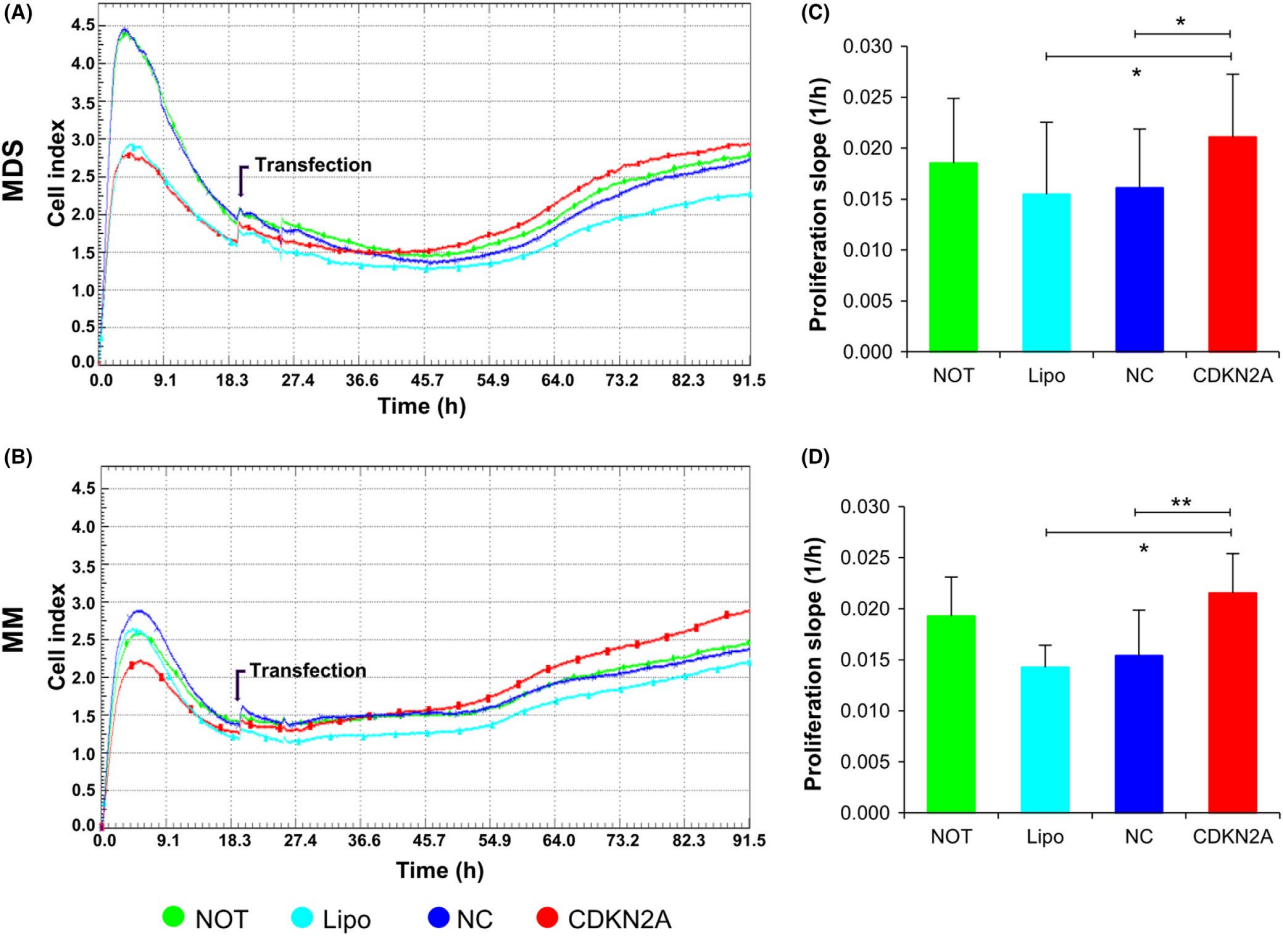
Real‐time monitoring of proliferation after CDKN2A knockdown (red line) using the xCELLigence assay compared with untreated (NOT, green), lipofectamine treated (Lipo, light blue) and negative control (NC)‐siRNA transfected (blue) bone marrow mesenchymal stromal cells (BM‐MSC) from myelodysplastic syndrome (MDS) and multiple myeloma (MM) for 72 hrs. Representative data by three technical replicates (A, B) and the mean and SE from the six independent donors (C, D). *P < .05, ** *P* < .01

Additional *CDKN2A* KD experiments were performed in MDS‐MSCs and MM‐MSCs. The cell index was enhanced in patient's BM‐MSCs after treated using siRNA targeting *CDKN2A* compared with untreated, lipofectamine treated and negative control‐siRNA transfected cells (Figure 5A,B). The average slope of growth curves during 3 days was highest in *CDKN2A* KD MSCs (Figure 5C,D). This indicated that patient's BM‐MSCs showed improved proliferation activity after downregulation of *CDKN2A*.

## 4 DISCUSSION

In this work, we evaluated characteristics of haematopoietic microenvironment in two types of haematologic malignancies and compared their characteristics. We selected MDS and MM to explore the similarity and difference of their characteristics because they represent myeloid and lymphoid neoplasms, respectively. They share similar nature such as late‐onset age and disease progression from low‐risk to high‐risk. Patient's BM‐MSCs exhibited a much lower proliferation activity, shorter telomere length and lower *CXCL12* expression compared to normal BM‐MSCs. When analyses were limited in patients, age and telomere length were not different according to degree of proliferation nor between disease category. We postulated that age was associated with physiological senescence of BM‐MSCs including telomere shortening, decreased proliferation activity and haematopoiesis support function rather than premature impairment of proliferation which was seen in patients.^19^, ^20^, ^21^, ^22^ Interestingly, *CXCL12* expression was low in poor proliferating MSCs, which functions HSC maintenance and regulation including quiescence and the ability to induce multilineage reconstitution.^23^ Downregulation of the haematopoiesis associated genes was also observed in BM‐MSCs primed by MDS and MM cell lines, which could result in multilineage peripheral blood cytopenia in patients. Further studies will be needed to define the correlation among age and *CXCL12* expression with haematopoietic function in patients.^24^, ^25^, ^26^

Global gene expression profile by microarray provided significant molecular pathways to explain the impaired proliferation activity in patient's BM‐MSCs including upregulation of important genes in ERK1/ERK2 cascade and MAPK cascade. ERK activity is known to be correlated with increased β‐galactosidase activity and induction of classical senescence‐associated genes.^27^ More recently, MAPK signalling pathway has been shown to be involved in the mechanism of BM‐MSC apoptosis.^28^ Regarding downregulation of genes functioning in β‐catenin‐TCF complex assembly, MDS‐MSCs exhibited decreased canonical WNT^29^ and β‐catenin mRNA expression.^30^ In MM, a soluble inhibitor of canonical WNT signalling, DKK1, was secreted by malignant cells which induced degradation of β‐catenin in BM‐MSCs.^31^, ^32^ We also showed that genes involved in “positive regulation of WNT signalling pathway” were downregulated in IM‐9‐primed BM‐MSCs which may contribute to premature senescence and increased apoptotic and/or necrotic fraction in patient's BM‐MSCs.^33^

Interestingly, changes in the proliferation activity of BM‐MSCs were closely associated with differentiation potential. We observed reduced osteogenic potential in both MDS‐MSCs and MM‐MSCs.^34^, ^35^ Premature exhaustion of MDS‐MSCs was combined with downregulation of genes regulating stemness and osteogenic differentiation.^36^ MM‐MSCs also had impaired osteogenic potential resulting from a crosstalk with myeloma cells through stimulate production of DKK1 and interleukin‐6^31^ or through changes in microRNAs.^37^, ^38^ In addition, it was found that genes regulating angiogenesis were upregulated in both MDS‐MSCs and MM‐MSCs. This finding was strengthened by enrichment of genes regulating angiogenesis in SKM1‐ and IM‐9‐primed BM‐MSCs. These genetic alterations might increase the risk of bone loss and neovascularization in MDS and MM patients which correlated with disease progression and prognosis.^11^, ^39^, ^40^, ^41^ Hence, it is reasonable to consider anti‐angiogenic drugs as a priority for targeting BM microenvironment.^24^, ^42^, ^43^

Although MDS‐MSCs and MM‐MSCs underwent similar changes, there existed some differences depending on disease category. MM‐MSCs showed decreased adipogenic potential while MDS‐MSCs did not.^44^ Besides, gene expression profile utilizing microarray analysis revealed significant enrichment of neuron development and cardiogenic differentiation in MDS‐MSCs and MM‐MSCs, respectively. Recent studies have demonstrated an increase of nestin expressing stromal cells in some MDS and AML.^45^, ^46^, ^47^ MDS‐MSCs might have obtained neural stem cell nature to show more tendency to neurogenic differentiation in our experiment. Previously, it has been reported that stromal precursor antigen‐1 (STRO‐1)‐positive colony‐forming MSCs are increased in MM patients^48^ and STRO‐1 can lead to increased expression of cardiovascular‐relevant cytokines and enhanced trophic activity.^49^ Taken together with results from in vitro differentiation and ex vivo coculture, we postulate that BM‐MSCs undergo changes depending on disease category as a result of cellular stress induced by malignant cells through direct contact and/or secretion.

BM‐MSCs did not harbour the same genetic mutations present in malignant cells. However, we detected an *NF1* mutation in MDS‐MSCs at P3. *NF1* gene encodes neurofibromin 1 that plays a crucial role in modulating MSC differentiation into osteoblasts. Therefore, the skeletal abnormalities, such as osteoporosis seen in neurofibromatosis patients with *NF1* mutation might be caused by defect in osteogenic differentiation through activation of Ras/MAPK.^50^, ^51^ It is interesting that mutant allele burden increased during passaging. Somatic mutations were accumulated in MSCs by passage dependent manner due to replication stress^52^ or they were present at early passage of BM‐MSC in AML.^4^ Although we should not over interpret this result obtained from one patient, it is worthy to explore the meaning of mutations occurred in patient's BM‐MSCs.

We obtained insight that although most patients’ BM‐MSCs fell in senescence status, the degree was various. About one third of patient’s BM‐MSCs stopped to proliferate before P6. A similar proportion failed to be cultured. It is notable that there was difference in degree of proliferation activity between MSCs from low‐risk and high‐risk group. Previous studies have shown that there is a great difference between high‐risk MDS‐MSC and low‐risk MDS‐MSC in the immunoregulatory functions.^53^ In plasma cell dyscrasias, disease evolution and progression is independently affected by the biology of the surrounding BM niche.^54^ Numerous observations indicate that an altered BM microenvironment provides a nurturing niche that sustains haematologic malignancy and might even contribute to the emergence and evolution of malignant clones.^55^ Specifically, we found that *CDKN2A* expression was significantly increased in poorly proliferating MSCs and correlated with PDT. These findings led us to perform *CDKN2A* IHC on BM samples whose MSCs were not cultured. IHC showed that *CDKN2A* immunoreactivity was markedly increased in those samples. *CDKN2A* was induced by cellular stress through CDKN2A‐RB pathway. If stress persisted, sustained activation of *CDKN2A* became engaged upon irreversible senescence.^56^, ^57^ It is noteworthy that patient’s BM‐MSCs revealed improved proliferation activity after *CDKN2A* KD. Downregulation of p16^INK4a^ using siRNA targeting *CDKN2A* increased cell division during clonal expansion^58^ and neural stem cell self‐renewal depended on repression of *CDKN2A*.^59^ Taken together, we postulated that *CDKN2A* is a good candidate of therapeutic target to control BM microenvironment because early senescence is reversible through de‐induction of *CDKN2A*.^60^ However, a larger study using BM‐MSCs derived from a more homogeneous population of patients is needed in order to clarify the contribution of *CDKN2A* and other markers in the premature senescence of BM‐MSCs in MDS and MM. Moreover, the addition of anti‐angiogenic drugs in *ex‐vivo* co culture experiments will help clarify the role of this targeted therapy in improving the haematopoietic support of BM‐MSCs.

## 5 CONCLUSIONS

Our results highlighted that BM‐MSCs from MDS and MM patients underwent similar functional alteration via common molecular mechanisms to induce cellular senescence, cell death and neovascularization. Moreover, there were different alterations between disease categories, including decreased adipogenesis and tendency for cardiomyogenesis in MM‐MSCs, and tendency for neurogenic differentiation in MDS‐MSCs. This was the first study to demonstrate that the degree of alteration is different for each patient and the value of *CDKN2A* expression can excellently represent the impaired proliferation activity of patient's BM‐MSCs. Further studies are required to specify the functional and/or molecular alteration that might be therapeutic targets as well as recovery indicators.

## CONFLICTS OF INTEREST

The authors declare that they have no competing interests.

## AUTHOR CONTRIBUTIONS

YK and MK involved in conceptualization and design; S‐SP, Y‐JK and C‐KM provided patient data and samples; HC, JK, AK and DK involved in experiments, collection and assembly of data; HC, JMK, MK and YK involved in data analysis and interpretation; HC, YK and MK edited and wrote the manuscript.

## Supporting information

Figure S1‐S7

## Data Availability

The data that support the findings of this study are available from the corresponding author upon reasonable request.
