# Supplementary material for: Common and different alterations of bone marrow mesenchymal stromal cells in myelodysplastic syndrome and multiple myeloma
Source: Cell Prolif. 2020 May 5;53(5):e12819. doi: 10.1111/cpr.12819 (PMC7260074; doi:10.1111/cpr.12819)
Supplement: Supplementary file 1 — Figure S1‐S7 [file CPR-53-e12819-s001.doc]

Original Article

**Common and different alterations of**

**bone marrow mesenchymal stromal cells in myelodysplastic syndrome and multiple myeloma**

Hayoung Choi,^1,2^ Yonggoo Kim,^1,3^ Dain Kang,^1^ Ahlm Kwon,^1^ Jiyeon Kim,^1^ Jung Min Kim,^4^ Sung-Soo Park^5^, Yoo-Jin Kim^5^, Chang-Ki Min^5^ and Myungshin Kim^1,2,3^*

^1^ Catholic Genetic Laboratory Center, Seoul St. Mary’s Hospital, College of Medicine, The Catholic University of Korea, Seoul, Republic of Korea

^2^ Department of Biomedicine & Health Sciences, Graduate School, The Catholic University of Korea, Seoul, Korea

^3^ Department of Laboratory Medicine, College of Medicine, The Catholic University of Korea, Seoul, Republic of Korea

^4^ Heimbiotek, Inc., Gyeonggi-do, Republic of Korea

^5^ Department of Hematology, Leukemia Research Institute, Seoul St. Mary's Hematology Hospital, College of Medicine, The Catholic University of Korea, Seoul, Republic of Korea

***** Correspondence: [microkim@catholic.ac.kr](mailto:microkim@catholic.ac.kr); Tel: +82-2-2258-1645; FAX: +82-2258-7966

**SUPPLEMENTAL DATA**

**Table S1.** Association between disease state and proliferation activity of bone marrow mesenchymal stromal cells in myelodysplastic syndrome and multiple myeloma.

|  | **Disease state** | |  |
| --- | --- | --- | --- |
| **Proliferation group** | **Low-risk** | **High-risk** | ***P*** |
| Continued to proliferate after P6 | 10 (25.0%) | 3 (7.5%) | 0.015 |
| Stopped to proliferate before P6 | 6 (15.0%) | 7 (17.5%) |  |
| Culture failure | 3 (7.5%) | 11 (27.5%) |  |

Low-risk, very low/low/intermediate by Revised International Prognostic System (IPSS-R) in myelodysplastic syndrome and stage I by International Staging System (ISS) in multiple myeloma; High-risk, IPSS-R high/very high IPSS-R and ISS stage II/III; P6, passage 6.


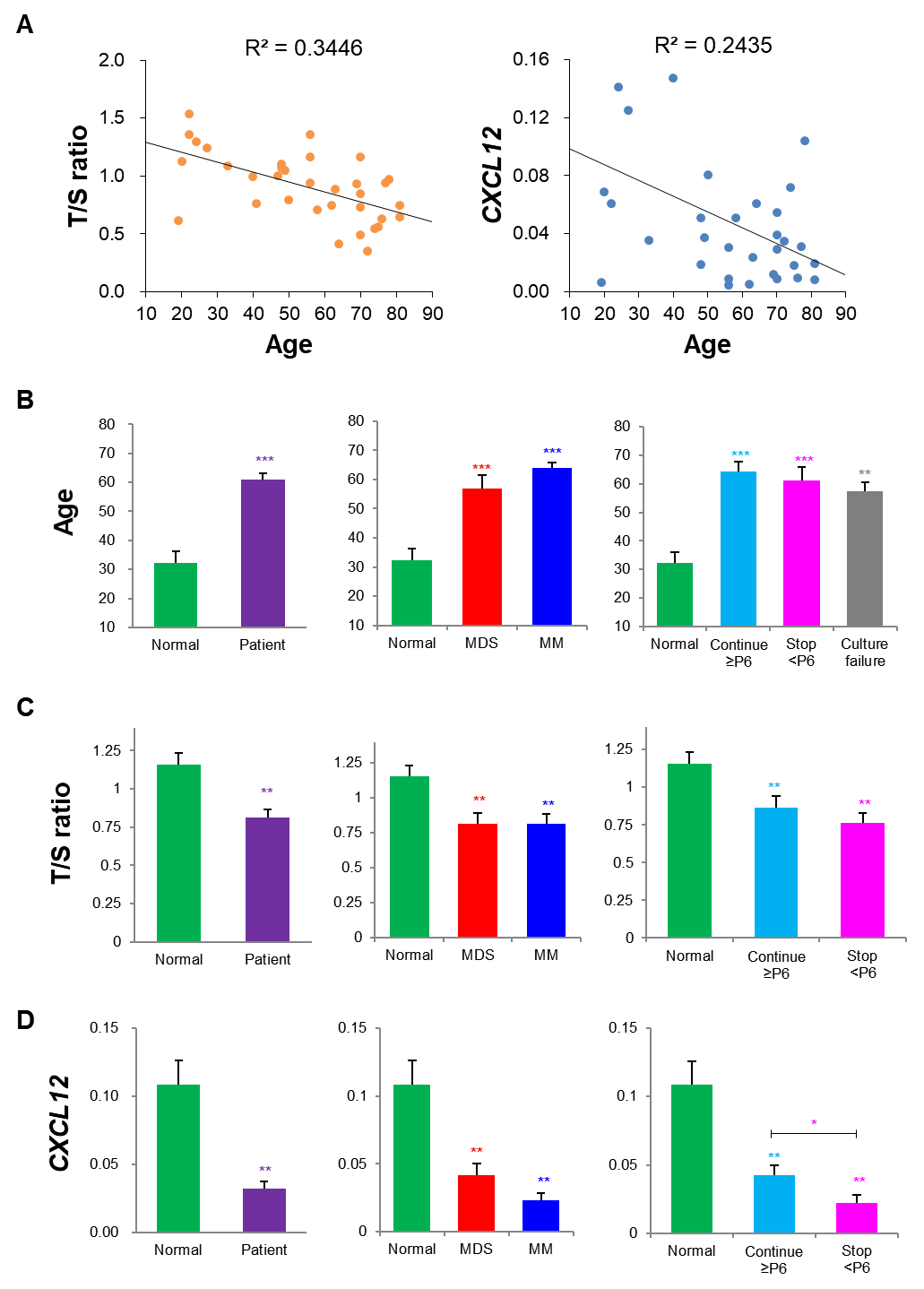


**Figure S1.** **(A)** Correlation between donor age and telomere-to-single copy gene (T/S) ratio (left) and *CXCL12* gene expression (right) of bone marrow mesenchymal stromal cells (BM-MSCs) from normal donors and patients including myelodysplastic syndrome (MDS) and multiple myeloma (MM) patients. Comparison of patient’s age **(B)**, T/S ratio **(C)** and *CXCL12* gene expression **(D)** according to disease category and proliferation activity of BM-MSCs.

**Figure S2.** Representative scatter plots showing increased apoptotic and/or necrotic fractions of bone marrow mesenchymal stromal cells (BM-MSCs) from myelodysplastic syndrome (MDS) multiple myeloma (MM) compared to normal BM-MSCs. 7-AAD, 7-Aminoactinomycin D.


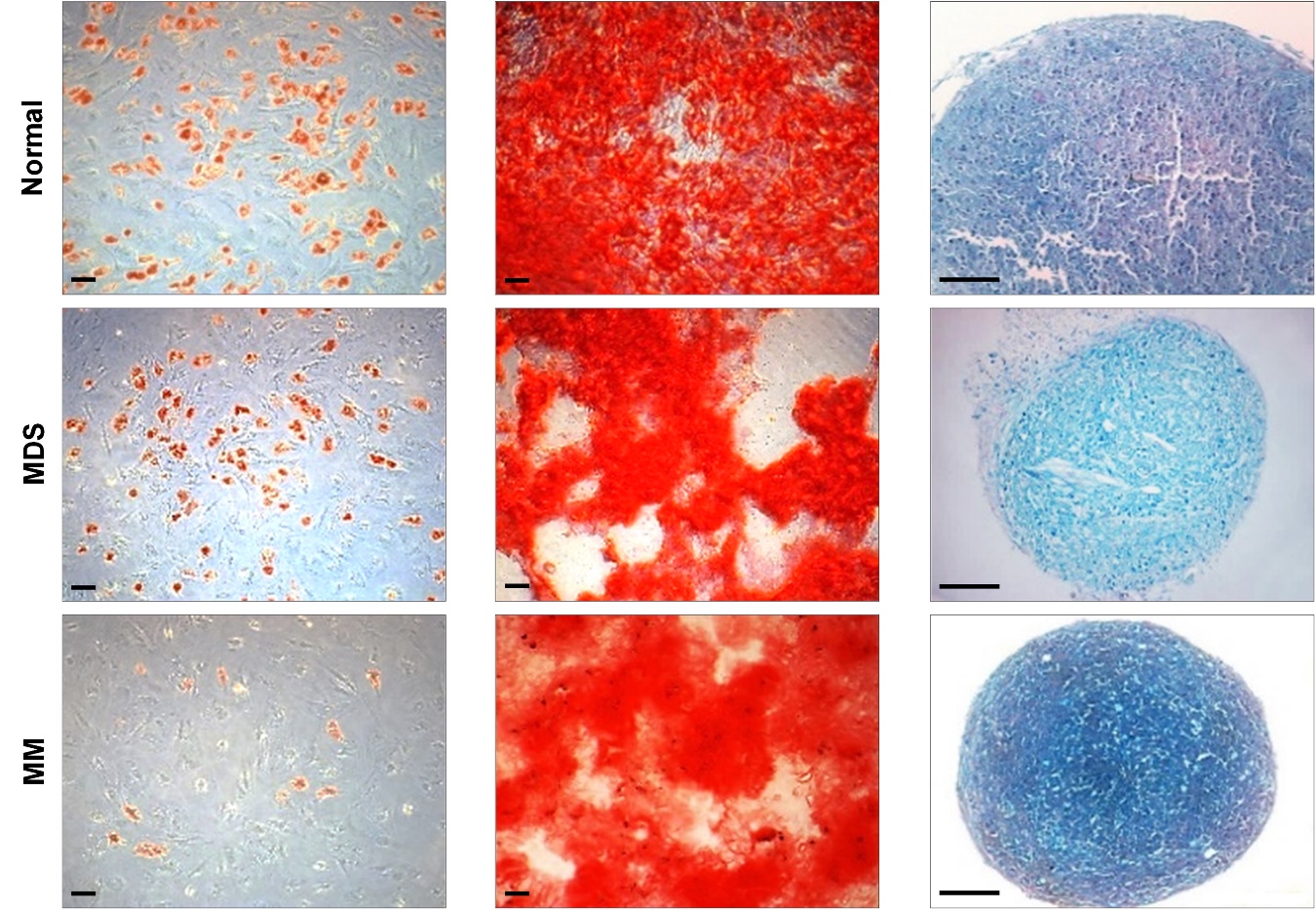


**Figure S3.** Representative microphotographs of Oil red O (left), Alizarin Red S (middle) and Alcian blue (right) staining of normal bone marrow mesenchymal stromal cells (BM-MSCs), myelodysplastic syndrome (MDS)-MSCs and multiple myeloma (MM)-MSCs cultured for 3 weeks in adipogenic, osteogenic and chondrogenic differentiation conditions, respectively. Both MDS-MSCs and MM-MSCs show reduced osteogenic potential against normal BM-MSCs. Reduced adipogenic potential is observed in MM-MSCs. Scale bars, 100 µm.


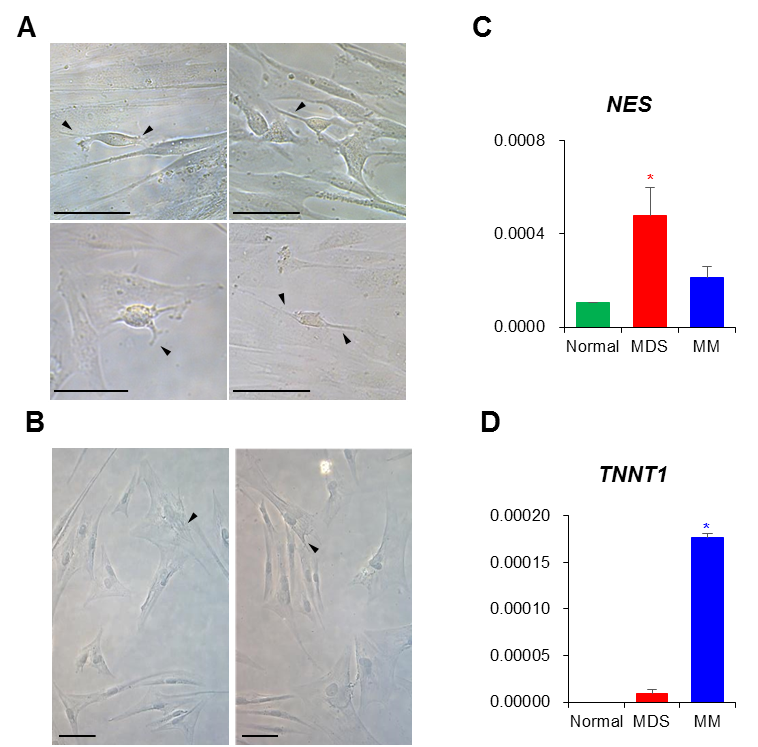


**Figure S4. (A)** Representative microphotographs after neurogenic differentiation of bone marrow mesenchymal stromal cells (BM-MSCs) from myelodysplastic syndrome (MDS). The arrows indicate cells with coarctate bodies and process-like extensions. **(B)** Morphologic changes after cardiomyegenic differentiation of MSCs from multiple myeloma (MM). The arrows indicate cells with increased granular contents and stress fibers **(C)** Comparison of *NES* and **(D)** *TNNT1* gene expression after neurogenic and cardiomyogenic differentiation. Scale bars, 100 µm.

**
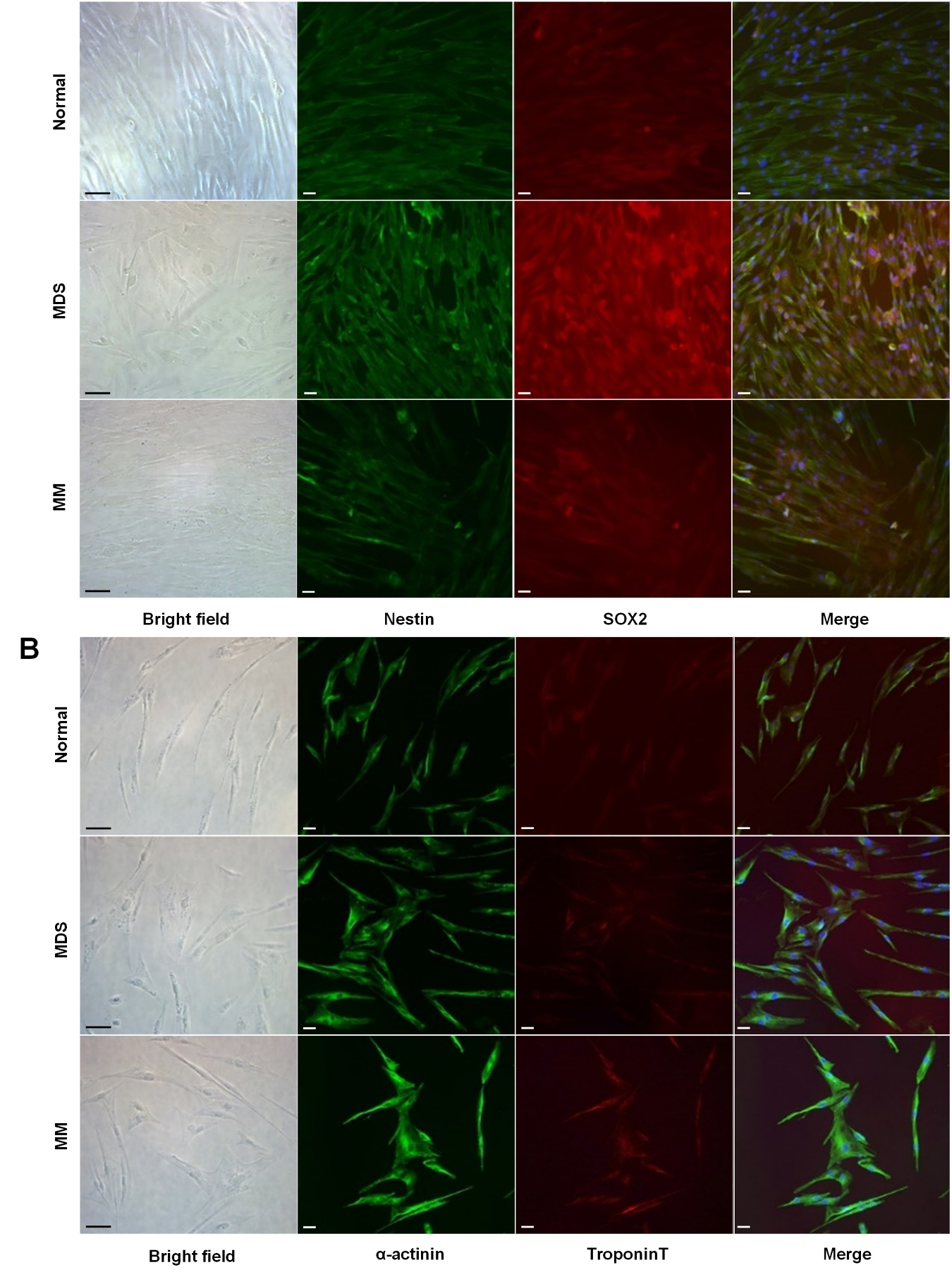
**

**A**

**Figure S5.** **(A)** Increased tendency for neurogenic differentiation of bone marrow mesenchymal stromal cells (BM-MSCs) from myelodysplastic syndrome (MDS) compared to those from multiple myeloma (MM) and normal. From the left, bright field, immunofluorescent staining of Nestin (green) and SOX2 (red), and merged image. Scale bars, 100 μm. **(B)** Increased tendency for cardiomyogenic differentiation of MM-MSCs. From the left, bright field, immunofluorescent staining of α-actinin (green) and cardiac Troponin T (red), and merged image. Scale bars, 100 μm.


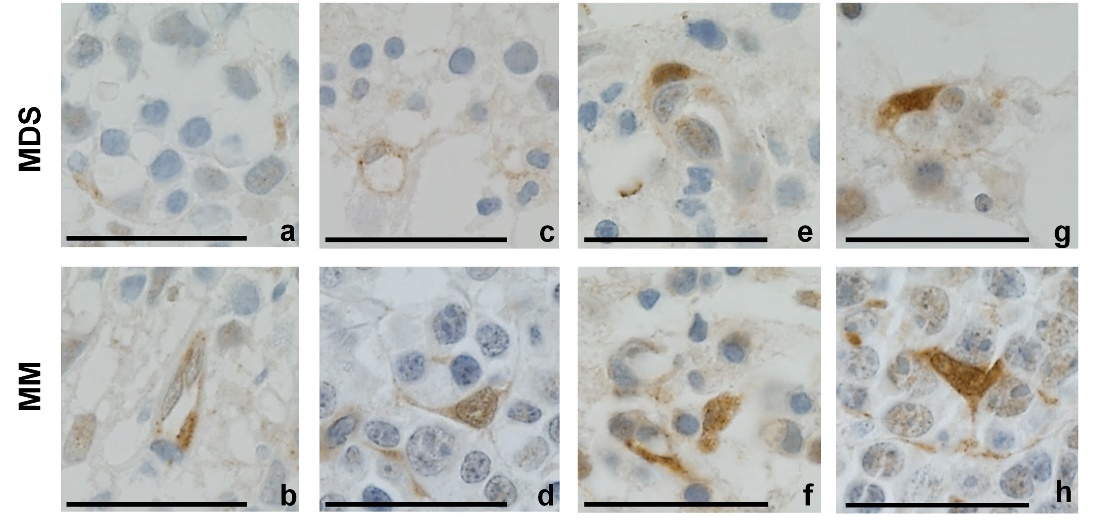


**Figure S6.** CDKN2A immunoreactive cells (stained brown) in bone marrow (BM) biopsy sections from myelodysplastic syndrome (MDS) and multiple myeloma (MM) patients. Majority are located near the vascular structure. Slight to moderate cytoplasmic (a, b) and nuclear (c, d) staining on perivascular cells in BM whose mesenchymal stromal cells (BM-MSCs) stopped to proliferate before passage 6. Intense cytoplasmic (f) and nuclear (e, g, h) staining of BM that failed in colony formation. Images were obtained with an Carl Zeiss Microscopy GmbH (Carl Zeiss) equipped with a ProgRes MF camera (JENOPTIK); original magnification, 1000x. Scale bars, 100 μm.


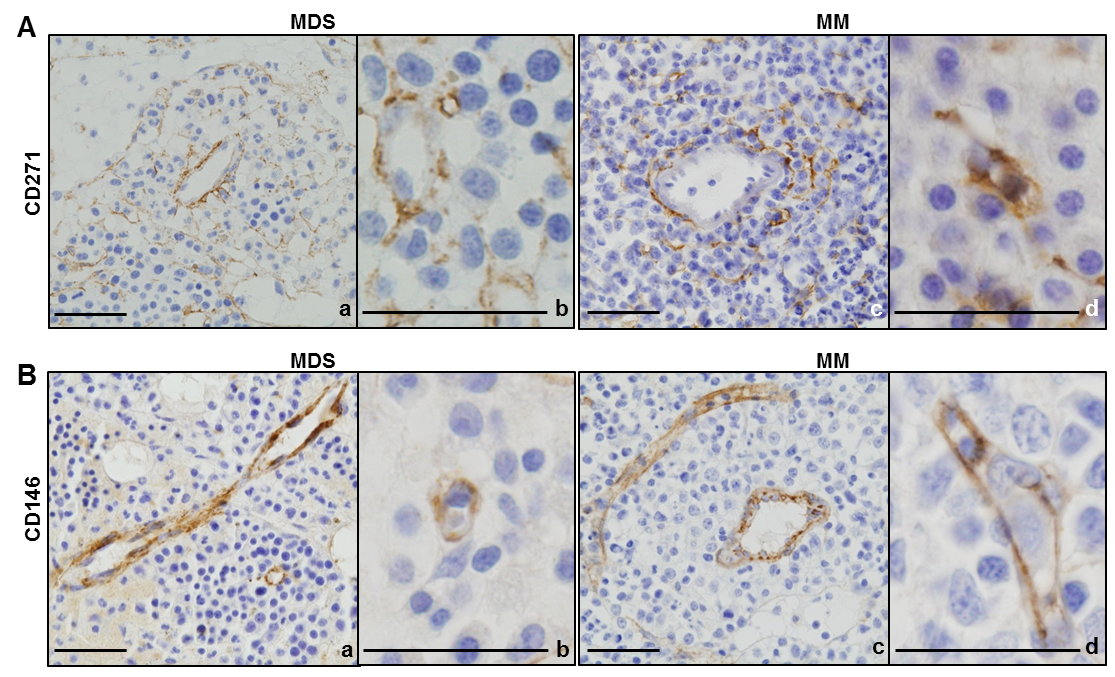


**Figure S7. (A)** Immunohistochemical staining of CD271 and **(B)** CD146 in bone marrow biopsy sections from MDS (a,b) and MM (c,d) patients. Images were obtained with an Carl Zeiss Microscopy GmbH (Carl Zeiss, Jena, Germany) equipped with a ProgRes MF camera (JENOPTIK, Jena, Germany); original magnification, 400x. Scale bars, 100 μm.
